# Reduced mammary gland carcinogenesis in transgenic mice expressing a growth hormone antagonist

**DOI:** 10.1054/bjoc.2001.1895

**Published:** 2001-08

**Authors:** M Pollak, M-J Blouin, J-C Zhang, J J Kopchick

**Affiliations:** Cancer Prevention Research Unit of the Jewish General Hospital and McGill University, Montreal, Quebec, H3T 1E2, Canada; Department of Biomedical Sciences, Edison Biotechnology Institute, Ohio University, Athens, Ohio, 45701, USA

**Keywords:** IGF-I, growth hormone antagonist, breast cancer, birthweight, prevention, risk, DMBA

## Abstract

Several reports have provided evidence that body size early in life is positively correlated with risk of subsequent breast cancer, but the biological basis for this relationship is unclear. We examined tumour incidence in transgenic mice expressing a growth hormone (GH) antagonist and in non-transgenic littermates following exposure to dimethylbenz[*a*]anthracene (DMBA), a well characterized murine mammary gland carcinogen. The transgenic animals had lower IGF-I levels, were smaller in terms of body size and weight, and exhibited decreased tumour incidence relative to controls. The demonstration that both body size early in life and breast cancer incidence are influenced by experimental perturbation of the GH–IGF-I axis in a transgenic model provides evidence that variability between individuals with respect to these hormones underlies the relationship between body size early in life and breast cancer risk observed in epidemiological studies. © 2001 Cancer Research Campaign http://www.bjcancer.com


*British Journal of Cancer* (2001) **85**(3), 428–430
© 2001 Cancer Research Campaign
doi: 10.1054/ bjoc.2001.1895, available online at http://www.idealibrary.com on IDEAL®

http://www.bjcancer.com

Short Communication

### Reduced mammary gland carcinogenesis in transgenic mice expressing a growth hormone antagonist

**M Pollak[1], M-J Blouin[1], J-C Zhang[1] and JJ Kopchick[2]**

[1]Cancer Prevention Research Unit of the Jewish General Hospital and McGill University, Montreal, Quebec, H3T 1E2, Canada;
[2]Edison Biotechnology Institute and Department of Biomedical Sciences, Ohio University, Athens, Ohio, 45701, USA

**Summary** Several reports have provided evidence that body size early in life is positively correlated with risk of subsequent breast cancer, but the biological basis for this relationship is unclear. We examined tumour incidence in transgenic mice expressing a growth hormone (GH) antagonist and in non-transgenic littermates following exposure to dimethylbenz[*a*]anthracene (DMBA), a well characterized murine mammary gland carcinogen. The transgenic animals had lower IGF-I levels, were smaller in terms of body size and weight, and exhibited decreased tumour incidence relative to controls. The demonstration that both body size early in life and breast cancer incidence are influenced by experimental perturbation of the GH–IGF-I axis in a transgenic model provides evidence that variability between individuals with respect to these hormones underlies the relationship between body size early in life and breast cancer risk observed in epidemiological studies. © 2001 Cancer Research Campaign http://www.bjcancer.com

**Keywords**: IGF-I; growth hormone antagonist; breast cancer; birthweight, prevention; risk; DMBA

Insulin-like growth factor-I (IGF-I) is a peptide with both mitogenic and anti-apoptopic properties. Unlike other growth factors, it has characteristics of both a tissue growth factor and an endocrine hormone (Jones and Clemmons, 1995; Rajaram et al, 1997). Circulating IGF-I levels are subject to complex physiological regulation, and vary considerably between normal individuals (Harrela et al, 1996). Most circulating IGF-I originates in the liver, and growth hormone is a key up-regulator of hepatic IGF-I production. IGF bioactivity in tissues is not merely a function of circulating concentration; local expression of IGFs, IGF-binding proteins, and proteases that digest IGF-binding proteins are also important factors. Clinical and experimental evidence suggesting important roles of IGF physiology in carcinogenesis and neoplastic progression have been the subject of recent reviews (Burroughs et al, 1999; Khandwala et al, 2000; Pollak, 2000).

Several epidemiological studies provide evidence that circulating IGF-I level is related to breast cancer risk among premenopausal women (Peyrat et al, 1993; Bruning et al, 1995; Bohlke et al, 1998; Hankinson et al, 1998; Toniolo et al, 2000; Li et al, 2001). A recent report (Byrne et al, 2000) provides evidence that circulating IGF-I level is highly correlated with mammographic density, which has previously been shown to be related to breast cancer risk. Separate data also suggest a relationship between circulating IGF-I level and risk of other cancers (Barinaga, 1998; Chan et al, 1998; Maison et al, 1998; Wolk et al, 1998; Burroughs et al, 1999; Holly et al, 1999; Manousos et al, 1999; Yu et al, 1999; Holly and Smith, 2000; Shaneyfelt et al, 2000). The mechanism underlying the IGF-I-cancer risk relationship remains unclear, but may relate to increased epithelial cell turnover and/or increased cell survival in individuals with higher IGF-I tissue bioactivity (Cohen and Ellwein, 1990; Ng et al, 1997; Pollak et al, 1999; Pollak, 2000).

Separate research has provided evidence that body size or weight early in life is positively correlated with subsequent risk of breast cancer (Michels et al, 1996; Stavola et al, 2000; Kaijser et al, 2001). The biological basis for this observation has been the subject of considerable speculation (for example, Vatten, 1996; Stoll, 1997; Signorello and Trichopoulos, 1998) but has not been addressed in experimental work. In view of evidence that body size early in life is related to cord blood IGF-I level (Ong et al, 2000), we hypothesized that the reason that there is a relation between body size early in life and subsequent breast cancer risk is that both are influenced by IGF-I levels. IGF-I levels are known to vary substantially between normal individuals, and approximately 50% of this variation is attributable to genetic factors which have not yet been characterized (Harrela et al, 1996). To address this hypothesis, we studied the effect of experimental perturbation of the GH-IGF-I axis in a transgenic mouse model on experimental mammary gland carcinogenesis.

Transgenic mice that constitutively express a bovine (Chen et al, 1990, 1991) or human (Chen et al, 1994) GH antagonist have been produced. In the present study, we examined tumour incidence in GH antagonist transgenic (GHA-tg) mice and in non-transgenic littermates in the standard dimethylbenz[*a*]anthracene (DMBA) breast carcinogenesis model in order to determine if an intervention targeted specifically at the GH–IGF-I axis would influence breast carcinogenesis.

28 8-week-old female GHA-tg mice (Chen et al, 1990) and 26 control littermates of the same age were given, by gavage, DMBA (70 µg $g^{-1}$ body weight) resuspended in peanut oil. The gavages were administrated once a week for 6 weeks. After the last gavage, tumour appearance was monitored weekly and tumours were measured with a calipre. Mice with tumours were sacrificed when tumours reached~1 cm in diameter or 39 weeks after DMBA treatment. The mice were weighed, measured and blood was collected

*Received 22 December 2000*
*Revised 24 April 2001*
*Accepted 26 April 2001*

*Correspondence to:* M Pollak

**Table 1** Characteristics of control and growth hormone antagonist transgenic mice

| | Body length (cm) | Body weight (g) | Circulating IGF-I concentration (ng ml$^{-1}$) | Percentage without tumour at 39 weeks (%) |
|---|---|---|---|---|
| Control mice | 9.5 ± 0.5 | 31.0 ± 2.6 | 186.9 ± 8.8 | 31.6 |
| Growth hormone antagonist transgenic mice | 7.6 ± 0.2 | 17.7 ± 3.2 | 104.5 ± 2.6 | 68.2 |
| *P* value | < 0.005 | < 0.0001 | < 0.0001 | < 0.001 |

The results are expressed as the mean ± SEM.

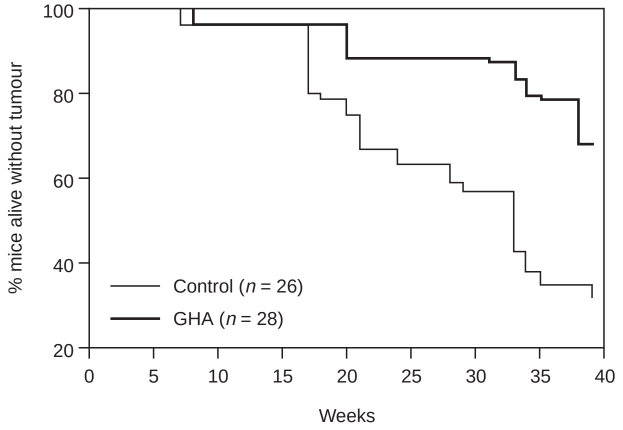

**Figure 1** Tumour incidence in growth hormone antagonist (GHA) transgenic mice vs. control mice following DMBA exposure. During the 39 weeks following DMBA treatment, tumour appearance was monitored as described in the text.

by cardiac puncture at time of sacrifice. Serum was frozen and subsequently assayed using an ELISA for rodent IGF-I (reagents from Diagnostic System Limited, Webster, Texas). Mice were handled in accordance with a protocol approved by McGill University and Lady Davis Institute animal care committees, and in accordance with the UKCCCR guidelines, and were maintained in a temperature-controlled environment with diurnal cycle of 12 hours of light and 12 hours of darkness, on an ad libitum diet (UKCCCR, 1998). Statistical tests (Student's *t*-test) were two-sided; *P* values of < 0.05 were considered to be significant.

The GHA-tg mice had lower levels of circulating IGF-I than control littermates (Table 1). GHA-tg mice were significantly smaller and lighter than non-transgenic animals, as previously reported (Chen et al, 1990). The GHA-tg female mice had grossly normal mammary glands, and normal ductal and stromal architecture on routine microscopy. Lactational performance of these mice was normal.

Tumour incidence was substantially reduced in GHA-tg mice compared to control mice (Table 1, Figure 1). The difference between the tumour incidence curves was highly significant ($P < 0.001$). The tumours were breast adenocarcinomas, as previously reported with the DMBA model (Russo and Russo, 1996).

Epidemiological studies have provided evidence that individuals with circulating IGF-I levels at the high end of the normal range have higher risk for premenopausal breast cancer than individuals with IGF-I levels at the low end of the normal range (Peyrat et al, 1993; Bruning et al, 1995; Bohlke et al, 1998; Hankinson et al, 1998; Toniolo et al, 2000; Li et al, 2001).

Separate evidence suggests that body size and weight early in life are related to subsequent breast cancer risk (Michels et al, 1996; Stavola et al, 2000; Kaijser et al, 2001). We show in a transgenic model that the phenotype associated with expression of a GH antagonist includes not only reduced body size and reduced circulating IGF-I level, but also reduced susceptibility to DMBA-induced mammary gland carcinogenesis. This suggests that inter-individual variations in the GH–IGF-I axis have important influences on both birthweight and breast cancer risk, and thus may explain at least in part the association between birthweight and breast cancer risk.

Our study design does not allow us to determine if there is a critical time at which IGF-I levels influence risk. It is known that a significant component of the inter-individual variation in IGF-I levels is genetically determined (Harrela et al, 1996), and it is possible that the influence of IGF-I on cancer risk operates through effects on epithelial cell renewal dynamics throughout life, including prenatal life, the period of pubertal breast development, and young adulthood.

The recent description of the safety and IGF-I lowering action of a GH antagonist in acromegalics (Trainer et al, 2000), together with our experimental results, raise the possibility that targeting the GH–IGF-I axis may represent a novel approach for cancer risk reduction for individuals with IGF-I levels at the high end of the normal range. This would be particularly relevant to the challenge of risk reduction for individuals who have known cancer risk factors such as carcinogen exposure, a germ line mutation in a cancer predisposition gene, or mutagen sensitivity, whose risk may be amplified by high IGF-I levels (Wu et al, 2000). It remains to be determined, however, if reduction of IGF-I levels from the high to the low end of the normal range in adulthood would in fact reduce IGF-I related cancer risk. It is conceivable that IGF-I levels in middle age are related to cancer risk only because they represent a surrogate for levels at some early critical period, such as puberty, at which time they influence risk. If this is the case, then interventions later in life will have little effect. Animal models of this issue are difficult due to species differences in growth, development and puberty. Nevertheless, early work involving perturbation of the GH–IGF-I axis with somatostatin analogues subsequent to carcinogen exposure in murine mammary carcinogenesis systems (Pollak and Schally, 1998) does provide evidence for reduction mammary cancer incidence even if treatment is deferred and should provide a foundation for further research.

### ACKNOWLEDGEMENTS

The work was supported by grants from Canadian Breast Cancer Research Initiative (to MP) and by the State of Ohio's Eminent Scholar Program including a grant to JJK from Melton & Lawrence Goll and by Sensus Corp.

### REFERENCES

Barinaga M (1998) Study suggests new way to gauge prostate cancer risk. *Science* **279**: 475

© 2001 Cancer Research Campaign

Bohlke K, Cramer DW, Trichopoulos D and Mantzoros CS (1998) Insulin-like growth factor-I in relation to premenopausal ductal carcinoma in situ of the breast. *Epidemiology* **9**: 570–573

Bruning PF, Van DJ, Bonfrer JM, Van NP, Korse CM, Linders TC and Hart AA (1995) Insulin-like growth-factor-binding protein 3 is decreased in early-stage operable pre-menopausal breast cancer. *Int J Cancer* **62**: 266–270

Burroughs KD, Dunn SE, Barrett JC and Taylor JE (1999) Insulin-like growth factor-I: a key regulator of human cancer risk? *J Natl Cancer Inst* **91**: 579–581

Byrne C, Colditz GA, Willett WC, Speizer FE, Pollak M and Hankinson SE (2000) Plasma insulin-like growth factor-I, insulin-like growth factor-binding protein-3 and mammographic density. *Cancer Res* **60**: 3744–3748

Chan JM, Stampfer MK, Giovannucci E, Gann PH, Ma J, Wilkinson P, Hennekens CH and Pollak M (1998) Plasma insulin-like growth factor-I and prostate cancer risk: a prospective study. *Science* **279**: 563–566

Chen WY, Wight DC, Wagner TE and Kopchick JJ (1990) Expression of a mutated bovine growth hormone gene suppresses growth of transgenic mice. *Proc Natl Acad Sci USA* **87**: 5061–5065

Chen WY, Wight DC, Mehta BV, Wagner TE and Kopchick JJ (1991) Glycine 119 of bovine growth hormone is critical for growth-promoting activity. *Mol Endocrinol* **5**: 1845–1852

Chen WY, Chen NY, Yum J, Washer TE and Kopchick JJ (1994) In vitro and in vivo studies of antagonistic effects of human growth hormone analogues. *J Biol Chem* **269**: 15892–15897

Cohen SM and Ellwein LB (1990) Cell proliferation in carcinogenesis. *Science* **249**: 1007–1011

Hankinson SE, Willett WC, Colditz GA, Hunter DJ, Michaud DS, Deroo B, Rosner B, Speizer FE and Pollak M (1998) Circulating concentrations of insulin-like growth factor-I and risk of breast cancer. *Lancet* **351**: 1393–1396

Harrela M, Koinstinen H, Kaprio J, Lehtovirta M, Tuomilehto J, Eriksson J, Toivanen L, Koskenvuo M, Leinonen P, Koistinene R and Seppala M (1996) Genetic and environmental components of interindividual variation in circulating levels of IGF-I, IGF-II, IGFBP-1, and IGFBP-3. J *Clin Invest* **98**: 2612–2615

Holly J and Smith GD (2000) Cancer and Insulin-like growth factor-I. *BMJ* **321**: 847–848

Holly JM, Gunnell DJ and Smith GD (1999) Growth hormone, IGF-I and cancer. Less intervention to avoid cancer? More intervention to prevent cancer? *J Endo* **162**: 321–330

Jones JI and Clemmons DR (1995) Insulin-like growth factors and their binding proteins: biological actions. *Endocr Rev* **16**: 3–34

Kaijser M, Lichtenstein P, Granath F, Erlandsson G, Cnattingius S and Ekbom A (2001) In utero exposures and breast cancer: a study of opposite-sexed twins. *J Natl Cancer Inst* **93**: 60–62

Khandwala HM, McCutcheon IE, Flyvbjerg A and Friend KE (2000) The effects of insulin-like growth factors on tumorigenesis and neoplastic growth. *Endocr Rev* **21**: 215–244

Li BD, Khosravi MJ, Berkel HJ, Diamandi A, Dayton MA, Smith M and Yu H (2001) Free insulin-like growth factor-I and breast cancer risk. *Int J Cancer* **91**: 736–739

Maison P, Balkau B, Simon D, Chanson P, Rosselin G and Eschwege E (1998) Growth hormone as a risk for premature mortality in healthy subjects: data from the Paris prospective study. *BMJ* **316**: 1132–1133

Manousos O, Souglakos J, Bosetti C, Tzonou A, Chatzidakis V, Trichopoulos D, Adami HO and Mantzoros C (1999) IGF-I and IGF-II in relation to colorectal cancer. *Int J Cancer* **83**: 15–17

Michels KB, Trichopoulos D, Robins JM, Rosner BA, Manson JE, Hunter DJ, Colditz GA, Hankinson SE, Speizer FE and Willett WC (1996) Birthweight as a risk factor for breast cancer. *Lancet* **348**: 1542–1546

Ng ST, Zhou J, Adesanya OO, Wang J, LeRoith D and Bondy CA (1997) Growth hormone treatment induces mammary gland hyperplasia in aging primates. *Nature Medicine* **3**: 1141–1144

Ong K, Kratzsch J, Kiess W, Costello M, Scott C and Dunger D (2000) Size at birth and cord blood levels of insulin, insulin-like growth factor I (IGF-1), IGF-II, IGF-binding protein-1 (IGFBP-1), IGFBP-3, and the soluble IGF-II/mannose-6-phosphate receptor in term human infants. The ALSPAC Study team. Avon Longitudinal Study of Pregnancy and Childhood. *J Clin Endocrinol Metab* **85**: 4266–4269

Peyrat JP, Bonneterre J, Hecquet B, Vennin P, Louchez MM, Fournier C, Lefebvre J and Demaille A (1993) Plasma insulin-like growth factor-1 (IGF-1) concentrations in human breast cancer. *Eur J Cancer* **29A**: 492–497

Pollak M (2000) Insulin-like growth factor physiology and cancer risk. *Eur J Cancer* **36**: 1224–1228

Pollak MN and Schally AV (1998) Mechanisms of antineoplastic action of somatostatin analogs. *Proc Soc Exp Biol Med* **217**: 143–152

Pollak M, Beamer W and Zhang J-C (1999) Insulin-like growth factors and prostate cancer. *Cancer and Metastases Reviews* **17**: 383–390

Rajaram S, Baylink DJ and Mohan S (1997) Insulin-like growth factor-binding proteins in serum and other biological fluids: regulation and functions. *Endo Rev* **18**: 801–831

Russo J and Russo IH (1996) Experimentally induced mammary tumors in rats. *Breast Cancer Research & Treatment* **39**: 7–20

Shaneyfelt T, Husein R, Bubley GJ and Mantzoros CS (2000) Hormonal Predictors of Prostate Cancer: A Meta-Analysis. *J Clin Oncol* **18**: 847–853

Signorello LB and Trichopoulos D (1998) Perinatal determinants of adult cardiovascular disease and cancer. *Scand J Soc Med* **26**: 161–165

Stavola BL, Hardy R, Kuh D, Silva IS, Wadsworth M and Swerdlow AJ (2000) Birthweight, childhood growth and risk of breast cancer in British cohort. *Br J Cancer* **83**: 964–968

Stoll BA (1997) Birthweight as risk factor for breast cancer. Lancet 349: 501–502

Toniolo P, Brunning PF, Akhmedkhanov A, Bonfrer JM, Koenig KL, Luknanova A, Short PA and Zeleniuch-Jacquotte A (2000) Serum insulin-like growth factor-I and breast cancer. *Int J Cancer* **88**: 828–832

Trainer PJ, Drake MB, Katznelson L, Freda PU, Herman-Bonert V, van der Lely AJ, Dimaraki EV, Stewart PM, Friend KE, Vance ML, Besser GM and Scarlett JA (2000) Treatment of acromegaly with the growth hormone-receptor antagonist Pegvisomant. *New Eng J Med* **342**: 1171–1177

United Kingdom Co-ordinating Committee on Cancer Research (UKCCCR) (1998) Guidelines for the Welfare of Animals in Experimental Neoplasion (Second Edition). *Br J Cancer* **77**: 1–10

Vatten L (1996) Can prenatal factors influence future breast cancer risk? *Lancer* **348**: 1531–1531

Wolk A, Mantzoros CS, Andersson S-O, Bergstrom R, Signorello LB, Lagiou P, Adami H-O and Trichopoulos D (1998) Insulin-like growth factor 1 and prostate cancer risk: a population-based, case-control study. *J Natl Cancer Inst* **90**: 911–915

Wu X, Yu H, Amos CI, Hong WK and Spitz MR (2000) Joint effect of insulin-like growth factors and mutagen sensitivity in lung cancer risk. *J Natl Cancer Inst* **92**: 737–743

Yu H, Spitz MR, Mistry J, Gu J, Hong WK and Wu X (1999) Plasma levels of insulin-like growth factor-I and lung cancer risk: a case-control analysis. *J Natl Cancer Inst* **91**: 151–156

*© 2001 Cancer Research Campaign*

